# The efficacy and safety of health qigong for anti-aging: Protocol for a systematic review and meta-analysis

**DOI:** 10.1097/MD.0000000000022877

**Published:** 2020-12-04

**Authors:** Peng Yu, Weihong Li, Han Li, Shuang Ouyang, Haiyang Cai, Jing Wu, Chaohui Tang, Qingsong Huang

**Affiliations:** a Basic Medical College, Chengdu University of TCM; b Hospital of Chengdu University of Traditional Chinese Medicine.

**Keywords:** aging, anti-aging, health qigong, systematic review

## Abstract

**Background:**

Aging is a phenomenon that human's physiology and psychology is progressive decline for natural environment. Health Qigong, as a convenient and effective exercise therapy,is widely used for anti-aging. However, there are no systematic reviews or meta-analysises to evaluate the efficacy and safety of Health Qigong on anti-aging.

**Methods:**

We will systematically search for 7 English databases(PubMed, Excerpta Medica Database, MEDLINE, Web of Science, Cochrane Library, SpringerLink, and WHO International Clinical Trials Registry Platform) and 4 Chinese databases(namely the China National Knowledge Infrastructure Database, the Wanfang Database, the Chinese Scientific Journal Database, and the Chinese BioMedical Literature Database) from their inceptions to August 2020. Randomized controlled trials (RCTs) using Health Qigong to anti-aging will be included. After the selection and extraction of eligible studies, a meta-analysis will be undertaken to assess the efficacy and safety of Health Qigong on anti-aging. Moreover, study selection, data extraction, and the evaluation of the methodological quality of trials will each be independently completed by at least 2 researchers. The Review Manager Software V.5.3 will be employed for meta-analysis to assess the risk of bias, data synthesis, and subgroup analysis.

**Results:**

This review will provide the latest knowledge and evidence on the efficacy and safety of Health Qigong for anti-aging through the analysis of various evaluation scales.

**Conclusion:**

The conclusion of this review will help clinicians provide effective exercise therapy for anti-aging.

**Registration number:**

INPLASY202090017

## 1 Introduction

Aging is a natural process which is generally along with a decline in the physiological function of the body, as a result of the susceptibility to age-associated diseases will be increased. For example, cardiovascular disease, dementia, osteoporosis, diabetes, hypertension, and stroke are age-induced diseases.^[1,2]^ Aging brings several diseases together will produce destructive effects on human society and breakdowns the entire health care system and economy.^[1,2]^

It is well known that aging is an inevitable physiological process, but we can delay senecence by various therapy.^[1]^ The potential mechanisms remain to be researched after many decades, and the oxidative stress and free radical accumulation theories stand out the most in the multiple theories related with aging. The antioxidant system declines as a growth of age, giving rise to destruction of the balance between radical oxygen species emergence and erasure issuing in oxidative cellular damage.^[3]^ Compared with other organs, postmitotic tissues such as the brain, heart and skeleton muscle are more susceptible to aging.^[4]^ Additionally oxidative stress, aging is also intimately related with issuing in structural and functional flaws in the immune system.^[5]^ The 1 of the primary feature of aging is piecemeal loss of cognition, with exhibition of descending logical thinking, memory and spacial abilities. The aging of cerebral is the primary cause of cognitive obstacle.^[6]^

The anti-aging interventions includes anti-aging medicine, herb, and Health Qigong. The anti-aging medicine according to mechanism include Calorie restriction mimetics, Activation of AMP protein kinase, Inhibition of growth hormone/insulin like growth factor-1 axis, Inhibition of mammalian target of rapamycin, Activation of the sirtuin pathway, Hormonal replacement, Gut microbiota, Vitamin D.^[7]^ The herb for anti-aging include Cistanches,^[8]^ Ganoderma Lucidum^[9]^ and so on. Health Qigong, containing Baduanjin, Wuqinxi, Yijinjing, Liuzijue and TaiChi, are traditional Chinese exercise, that are deemed to not only enhance the antioxidant capacity of the body but also regulate immune function. Previous researches have showed that Health Qigong has fine effects for anti-aging, including regulate mental and physical health,^[10–14]^ and age-associated diseases.^[15–17]^ Due to these strength of Health Qigong,doctors recommend using Health Qigong to increase vigor and vitality as well as prolong lifespan.

Although Health Qigong is widely applied to anti-aging increasing, system reviews and meta-analysis of Health Qigong are few. Therefore, we will perform a systematic review of randomized clinical trials summarizing the existing evidence and assessing the effectiveness and safety of Health Qigong for anti-aging.

## 2 Methods

The protocol of the systematic review and meta-analysis has been registered in the International Platform of Registered Systematic Review and Meta-analysis Protocols (INPLASY), the registration number is INPLASY202090017. It will be based on the guidelines of Preferred Reporting Items for Systematic Reviews and Meta Analysis (PRISMA-P).

### 2.1 Inclusion criteria for study selection

#### 2.1.1 Type of studies

Only the RCTs of Health Qigong for anti-aging will be included in this study, without placing the constraint on publication status and writing language. Studies without sufficient information about the randomized method or process, the animal mechanism studies, qualitative studies, uncontrolled trials and reviews and case reports will be excluded.

#### 2.1.2 Type of participants

Subjects aged 45 to 75 years, who do not exercising daily and have no major disease will be selected. There will be no restriction on gender, race, or nation.

#### 2.1.3 Type of interventions

Our systematic review and meta-analysis will be conducted based on the RCTs that solely apply Health Qigong intervention in the experimental group and placebo or other nonpharmacological therapies in the control group. The control group has the following forms: keep previous exercise habit, restrict any exercise etc. Health Qigong include Baduanjin, Wuqinxi, Yijinjing, Liuzijue and TaiChi.

#### 2.1.4 Type of outcome measurements

##### 2.1.4.1 Primary outcomes

The primary outcomes include the antioxidant capacity and the immune function. The antioxidant capacity are total antioxidant capacity, superoxide dismutase and Malondialdehyde. The immune function index are tumor necrosis factor-α counts, interferon -γ counts,IL-2 counts, IL-6 counts and the level of T cell subsets (Cluster of Differentiation [CD]3^+^,CD4^+^, CD8^+^, CD4^+^/CD8^+^).

##### 2.1.4.2 Secondary outcomes

The Secondary outcomes are cardio-cerebrovascular function and cognitive competence.

### 2.2 Search strategy

#### 2.2.1 Electronic searches

The following 7 English databases (PubMed, Excerpta Medica Database, MEDLINE, Web of Science, Cochrane Library, SpringerLink, and WHO International Clinical Trials Registry Platform) and 4 Chinese databases(namely the China National Knowledge Infrastructure Database, the Wanfang Database, the Chinese Scientific Journal Database, and the Chinese BioMedical Literature Database) will be investigated from their inception to August, 2020. The search strategy for selecting the fields of topic, title, or abstract was unique referring to the characteristics of databases.

The complete PubMed search strategy is in Table 1 and will be modified and used in other electronic databases.

**Table 1:** PubMed search strategy.

Number	Search terms
1	Health Qigong
2	Qigong
3	Baduanjin
4	Wuqinxi
5	Yjinjing
6	TaiChi
7	Tai Ji
8	TaiChi Chuan
9	Tai Ji quan
10	Liuzijue
11	Eight brocade
12	Five animals exercise
13	Six character formula
14	Or 1–13
15	aging
16	Anti-aging
17	Or 15–16
18	Random
19	Randomized
20	Randomly
21	Control
22	RCTs
23	Clinical trial
24	Or 18–23
25	14 and 17 and 24

RCT = randomized controlled trial.

#### 2.2.2 Searching other resources

We will investgate the preplanned, ongoing, and unpublished studies by searching Google Scholar, Baidu Scholar, the International Clinical Trials Registry Platform and the Chinese Clinical Trial Registry.

### 2.3 Data collection and analysis

#### 2.3.1 Selection of studies

**Figure 1:**
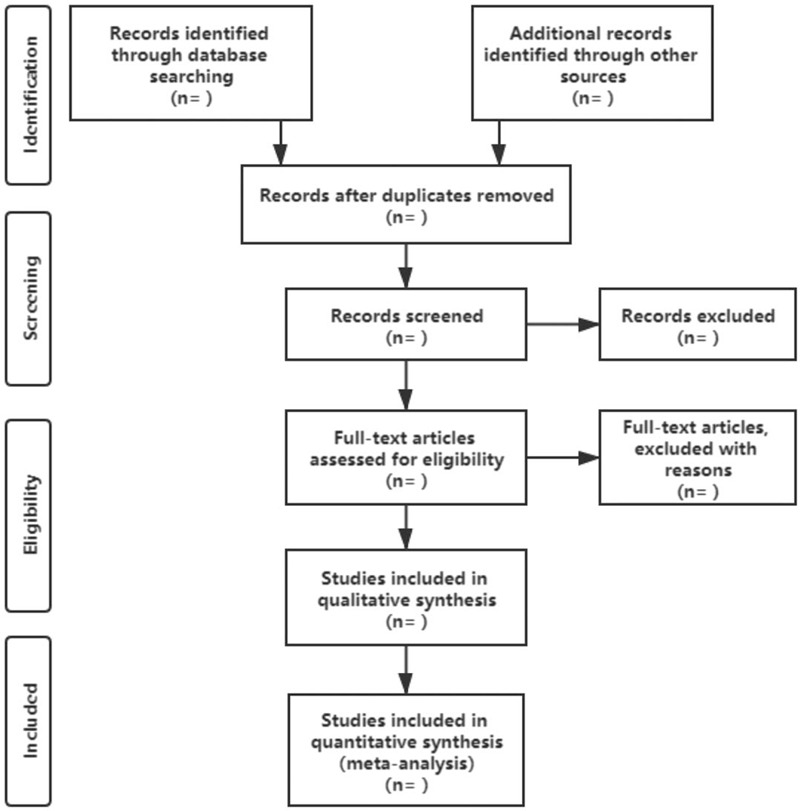
Flow diagram of study selection.

Two researchers(Han Li, Shuang Ouyang) will be individual into 2 groups, and each group will independently scan the titles and abstracts. After deleting repetitive and irrelevant articles, the 2 groups will set up 2 lists of potential studies which will be verified against each other by superintendent(Chaohui Tang) to make a preliminary list. Next authentication of eligible articles from the preliminary list will be accomplished by 2 researchers (Haiyang Cai, Jing Wu) through using the preplanned inclusion/exclusion criteria. When divergence appear another researchers(Peng Yu) will make a judgment. The selection procedure is fully described in a PRISMA flow chart (Fig. 1).

#### 2.3.2 Data extraction

Two researchers (Han Li, Shuanng Ouyang) will independently work for data extraction, they will collect the undermentioned data:

(1) basic characteristics, including the first author, publication year, sample size, trial location, age, geographic population, health status, duration and follow-up, frequency, intensity, Health Qigong style, control intervention.
(2) (2)primary outcomes and secondary outcomes as the information showed before will also be included, including the antioxidant capacity, the immune function and cardio-cerebrovascular function.

The results will be double-verified by a another researcher (Qingsong Huang). If the data is missing or unclear, the corresponding author will be contacted.

#### 2.3.3 Risk of bias assessment

We will according to the Cochrane Handbook(V.5.1.0)^[18]^ of Systematic Reviews of Interventions, and the risk of bias (ROB) of the included studies will be assessed by 2 researchers(Haiyang Cai, Jing Wu) independently using the Cochrane risk of bias assessment tool. The domains pertinent to ROB including random sequence generation, allocation concealment, blinding of participants and therapist, blinding of outcome assessment, incomplete outcome data, selective reporting, and other bias. The ROB for each domain will be rated as“low risk,” “high risk,” or “unclear risk.”

#### 2.3.4 Data synthesis

The meta-analysis will be performed by using the Review Manager V.5.3 (RevMan 5.3)software. We will describe the effect size with the risk Ration for dichotomous data, and the mean deference for continuous data.95% of the confidence interval will beused as an effective size for the combined analysis.

#### 2.3.5 Assessment of heterogeneity

We will calculate the heterogeneity by using the Chi-square and I^2^ statistic, which describes the percentage of variability in the effect estimates. I^2^ statistic (I^2^ values of 0% to 40% being interpreted as “might not be important”;30% to 60%: may represent moderate heterogeneity;50% to 90%: may represent substantial heterogeneity; and 75% to 100%: represents considerable heterogeneity). For more detailed explanation on potential heterogeneity among involved studies, we may also conduct subgroup analyses or meta-regression.

#### 2.3.6 Analysis of subgroups

If we have a sufficient number of RCTs for inclusion in the review, we will carry out the subgroups when the heterogeneity is high. Subgroup analysis will be carried out based on the type of control interventions, type of Health Qigong, exercise frequency and duration.

#### 2.3.7 Sensitivity analysis

We will conduct the sensitivity analysis to assess the robustness of bias on results and the sources of heterogeneity. We will delete the data of certain study 1 by 1 by comparing the recombined date with original date,and then focus on the changes merged size effects and heterogeneity.

#### 2.3.8 Assessment of reporting biases

If there are more than 10 studies are included in the review, we will build funnel plots to appraise the reporting bias. There are 2 results, it means no reporting bias existed when the funnel is symmetrical, or it means reporting bias when the funnel is dissymmetry.

#### 2.3.9 Confidence in cumulative evidence

We will assess the quality of evidence by using the GRADE system. The following 5 factors will be considered: risk of bias, inconsistency, directness, inaccuracy, and reporting bias. Four levels will be geaded of quality of evidence: high, moderate, low or very low.

## 3 Discussion

Aging is an unavoidable natural physiological process, but aging brings several diseases together will produce destructive effects on human society and breakdowns the entire health care system and economy.^[1,2]^ Hence, it is necessary to find effective non-drug therapies to anti-aging. Health Qigong, as a traditional Chinese excercise, has a remarkable effect on improving immune function and the antioxidant capacity of the body. However, there is still no systematic review clearly demonstrating the role of Health Qigong for anti-aging.

The results of this study will identify whether Health Qigong can delay senescence and related laboratory indicators of anti-aging, which will present an evidence for Health Qigong in applications. The small quantity of related literature and the high heterogeneity of different Health Qigong styles used in the research may attract limitations to this study. If there is enough randomized controlled double-blind experimental research, we will accomplish a subgroup analysis to identify specific Health qigong programs and exercise time. In addition, Health Qigong has a widely application in Asia, peculiarly China, but it is not well accepted in other parts of the world, so publication bias will also be considered.

In conclusion, this study will investigate the efficacy and safety of Health Qigong as a treatment of anti-aging.

## Author contributions

**Conceptualization**: Peng Yu, Weihong Li.

**Data curation**: Han Li, Shuang Ouyang.

**Formal analysis**: Haiyang Cai, Jing Wu.

**Methodology**: Peng Yu, Chaohui Tang, Qingsong Huang.

**Project administration**: Peng Yu, Weihong Li.

**Software**: Peng Yu.

**Supervision**: Chaohui Tang

**Validation**: Weihong Li

**Writing – original draft**: Peng Yu
